# The Contribution of the Internet to Promoting Mental Health for Older Adults: Cross-Sectional Survey in China

**DOI:** 10.2196/40172

**Published:** 2023-12-19

**Authors:** Weichao Chen, Mengjun Ding, Xiaoyan Wang

**Affiliations:** 1 Department of Editing and Publishing Hunan Normal University Changsha China; 2 Department of Statistics Hunan University Changsha China

**Keywords:** older adults, internet use, mental health, influence mechanism, participation in voluntary activities, subjective age, mobile phone

## Abstract

**Background:**

Health is an important topic for everyone and essential to high-quality economic and social development. Recently, some researchers have suggested that older adults’ internet use may have a health effect.

**Objective:**

This study specifically aims to clarify the relationship between internet use and the mental health of older adults, for which other surveys present contradictory results.

**Methods:**

Data were obtained from the China Longitudinal Aging Social Survey conducted in 2018. A total of 6648 participants were included. Mental health was assessed by the 12-item Center for Epidemiological Studies Depression Scale. Ordinary least squares regression was adopted to explore the relationship between internet use (independent variable) and the mental health of older adults. Robustness analysis, sensitivity analysis, and heterogeneity analysis were conducted in detail to verify the empirical result. A mediating effect analysis was further conducted to discover the effect mechanism between the dependent and independent variables.

**Results:**

It was found that internet use and smartphone use can significantly improve the mental health of older adults (ordinary least squares, β=.075; *P<*.001). After endogenous and robustness tests were conducted, the aforementioned conclusion remained robust. In particular, participation in voluntary activities played a mediating role in the relationship between internet use and the mental health of older adults. In addition, younger subjective age enhanced the positive effect of internet use on the mental health of older adults.

**Conclusions:**

Internet users showed higher levels of mental health among Chinese older adults. To improve the mental health of older adults, the government should not only cultivate the ability to use the internet but also encourage greater participation in voluntary activities among older adults.

## Introduction

### Background

Aging has become a common global public health concern owing to the dramatic increase in the number of older adults. As the country with the largest population of older adults in the world, China’s aging rate has exceeded the world average [1]. It is estimated that China’s population of older adults (those aged ≥60 y) will exceed 345 million by the end of 2030, posing huge challenges to the supply of medical and public services. Owing to its popularity, the internet has gradually changed from a way of entertainment to a way of production and life. At the same time, some scholars found that the internet continually penetrates the life of older adults worldwide and increasingly becomes a part of their life, which may have a health effect [2]. Although previous research has analyzed the direct impact of internet use on mental health, numerous studies have studied the inﬂuence mechanisms between internet use and the mental health of older adults. As for the indirect impact of internet use on the mental health of older adults, participation in voluntary activities may be a very important intermediary factor. Consequently, this study explores the relationship and influence mechanisms between internet use and the mental health of older adults in China.

There are 2 viewpoints on the relationship between internet use and mental health. According to the first viewpoint, internet use has a statistically signiﬁcant negative impact on mental health. Some researchers found that internet use is significantly associated with decreased time spent with friends and decreased local social networking, which increased loneliness and decreased various aspects of the quality of life [3], with weakly connected communication and web-based entertainment activities acting as substitutes [4]. Consequently, internet use further leads to loneliness and social marginalization among older adults [5]. In addition, internet use was signiﬁcantly and negatively associated with the life satisfaction of Chinese older adults owing to reduced perceptions of social justice and communication in real life [6].

According to the second viewpoint, internet use has a positive effect on mental health. On the basis of the health theory propounded by Grossman [7,8], internet use has a strong association with the mental health of older adults, and internet technology enhances health capital by meeting older adults’ personalized needs regarding mental health. Furthermore, some studies unveiled that internet use could improve the mental health of older adults by motivating them to increase their engagement in social activities [9,10]. Similarly, the more frequently older adults use the internet, the lower the possibility of having psychological problems, as was found in a study of older adults with chronic diseases in Shanghai, China [11]. Meanwhile, internet use has been shown to relieve older adults’ depression and anxiety [12].

Voluntary activities refer to activities that are freely chosen by individuals and carried out for free to benefit other people, groups, or organizations [13]. Previous studies have shown that age is an important factor that motivates people to engage in voluntary activities, implying that older adults are more likely to seek volunteering opportunities [14]; for example, some older adults choose volunteer work as an occupation after retirement [15].

Internet use can encourage older adults to engage in voluntary activities more frequently. In the case of youth groups, previous studies suggest that frequent internet use reduces the frequency of volunteering because time spent using the internet reduces the time that individuals can devote to volunteering [16]. By contrast, in the case of older adults, some scholars insist that internet use can strengthen participation in voluntary activities; for instance, the study by Mukherjee [17] found that older adults can overcome coordination problems in volunteer work by using the internet. In addition, Jin and Zhao [18] found that older adults who use the internet are more likely to participate in voluntary activities. However, Filsinger and Freitag [19] uncovered that internet use had a signiﬁcant negative impact on participation in voluntary activities.

Furthermore, participation in voluntary activities can improve the mental health of older adults. According to the activity theory, older adults who often participate in various activities tend to report higher levels of health [20]. Furthermore, some scholars have found that participation in voluntary activities is conducive to relieving depression among older adults, especially those aged >65 years [21]. Besides, Li and Ferraro [22] divided voluntary activities into formal voluntary activities and informal voluntary activities and found that participation in formal voluntary activities can help relieve depression, whereas participation in informal voluntary activities does not. This is mainly because informal volunteer work is less socially acceptable and requires a stronger sense of social responsibility than formal volunteer work, which may offset the positive effects of volunteering on depression. Moreover, Guo et al [23] analyzed data from the China Longitudinal Aging Social Survey (CLASS) conducted in 2014 and confirmed that participation in voluntary activities can significantly reduce the depression level of urban older adults in China but has no significant effect on the depression level of rural older adults.

Subjective age refers to how young or old individuals experience themselves to be and is associated with health status [24]. Subjective age is closely related to active aging, and the younger the subjective age, the more successful the active aging process [25]. Most older adults believe that their subjective age is less than their chronological age; the discrepancy between chronological age and subjective age in individuals’ personal dimensions might reﬂect their dissociation from old age in response to the exposure to negative age misconceptions and stigma [26]. Studies have shown that the younger an older adult feels, the more likely they will use the internet [27] and use it more frequently [28]. Participation in digital activities could enhance the sense of autonomy and self-efficacy of older adults, provide an endogenous driving force to help alleviate their mental loneliness, and help them to form a positive attitude and an enjoyable life experience [29]. In addition, regarding the relationship between subjective age and mental health, Teuscher [30] found that subjective age is significantly related to mental health factors such as optimism and self-efficacy. To be more specific, younger subjective age is associated with a lower risk of depression [31] and higher life satisfaction [32].

Studies have confirmed that individuals could easily obtain relevant information about voluntary activities through the internet, which in turn stimulated them to participate in offline voluntary activities [33]. The internet has an increasingly profound impact on older adults and has gradually changed their lifestyle [34].

### Objectives

On the basis of CLASS 2018 longitudinal data, this study enriches and expands previous research in terms of 3 aspects. First, the study divides internet use into 4 dimensions (whether to use the internet, the frequency of internet use, the number of web-based activities, and smartphone use), providing a multidimensional consideration of internet use connotation. Second, this study explains the mechanism of how internet use affects the mental health of older adults under the mediating role of participation in voluntary activities. Third and last, we explore whether subjective age plays a moderating effect between internet use and the mental health of older adults.

## Methods

### Data Sources

Data from CLASS 2018 were used to undertake a secondary analysis in this study. CLASS is a national longitudinal social survey organized and implemented by the Renmin University of China to comprehensively understand the basic situation of older adults. CLASS respondents are Chinese older adults aged ≥60 years, with a sample size of 11,418 in 2018. By eliminating the missing values for the variables that we were interested in, we obtained valid data of 6648 (58.22%) of the 11,418 respondents for the analysis.

### Variables and Measurements

#### Dependent Variable

In the CLASS questionnaire, a summary score of mental health is calculated using the Depressive Tendency Scale, a simpliﬁed version of the Center for Epidemiological Studies Depression Scale, with 12 questions covering aspects of daily mood, loneliness, sleep, suﬃciency, and the life status of older adults. Similar to previous studies [35,36], mental health was measured by 12-item general health questionnaire “Next, we would like to know your mood in the past week,” which lists 12 items (eg, “Did you feel in a good mood in the past week?”). For each activity (item), the respondents’ answers were coded as 1=often, 2=sometimes, and 3=never. The average score of 12 items was used to assess the mental health of older adults, with higher scores indicating a higher level of mental health. The Cronbach coefficient α of mental health scores was .861, indicating good internal consistency of the items in the scale.

#### Independent Variables

Following the study by Wang [37], internet use was measured by the single-item question “Do you use the internet (including computer and mobile internet)?” It is a binary variable, with 1 indicating smartphone use and 0 indicating other.

#### Mediating Variables

The mediating variable is participation in voluntary activities. Following the study by Chai and Guo [38], participation in voluntary activities was measured using 7 items: community security patrols; caring for other older adults or children; environmental sanitation protection; mediating neighborhood disputes; accompanying chat; volunteering services requiring professional skills; and caring for, and educating, the next generation. The frequency of participation in voluntary activities ranged from 1=never to 5=daily. In our analysis, we reassigned the values of these 7 activities: the value of participating in each activity was 1; otherwise, it was 0. The variable of *voluntary engagement* was the total for the 7 activities. Specifically, respondents who participated in any of these activities were defined as participating in voluntary activities, and those who did not participate in all activities were defined as not participating in voluntary activities.

#### Moderating Variable

Following the study by Hubley and Hultsch [39], the moderating variable was subjective age. We defined subjective age as the age one feels minus one’s chronological age, a more positive score indicating a more youthful subjective age. Specifically, the felt age was measured by the question “At what age does a person become old?” The chronological age is the older adult’s age based on their date of birth.

#### Control Variables

The control variables selected in this study included age, sex (0=female and 1=male), education level (0=uneducated, 1=primary school, 2=junior high school, and 3=high school and above), marital status (0=other and 1=married), hukou (household registration; 0=rural and 1=urban), work status (0=other and 1=employed), home ownership (0=does not own a home, 1=owns 1 home, and 2=owns ≥2 homes), social insurance (0=other and 1=has social insurance), and self-rated health (1=very unhealthy, 2=moderately unhealthy, 3=average, 4=moderately healthy, and 5=very healthy). Region of residence (east, central, or west) was represented by 2 dummy variables. The descriptive statistical analysis of all variables is presented in Table 1.

**Table 1:** Descriptive statistics of variables (n=6648).

Variable			Values
Mental health, mean (SD)			2.3122 (0.3439)
Participation in voluntary activities, mean (SD)			0.2373 (0.4254)
Internet use, n (%)			1488 (22.38)
Sex (male), n (%)			3310 (49.79)
Age (y), mean (SD)			70.8719 (7.0914)
**Hukou (household registration), n (%)**			
	Rural	3393 (51.04)	
	Urban	3255 (48.96)	
Marital status (married), n (%)			4631 (69.66)
Work status (employed), n (%)			1658 (24.94)
Social insurance (has social insurance), n (%)			5185 (77.99)
Self-rated health, mean (SD)			3.3206 (0.8626)
**Education, n (%)**			
	Uneducated	1921 (28.9)	
	Primary school	2430 (36.55)	
	Junior high school	1615 (24.29)	
	High school and above	682 (10.26)	
**Home ownership, n (%)**			
	Does not own a home	329 (4.95)	
	Owns 1 home	5822 (87.58)	
	Owns ≥2 homes	497 (7.48)	
**Region of residence, n (%)**			
	East	2505 (37.68)	
	Central	2434 (36.61)	
	West	1709 (25.71)	

### Ethical Considerations

The datasets generated and analysed during the current study are publicly available in the China Longitudinal Ageing Social Survey (CLASS) repository [40], and the authors received permission to use the data. CLASS was approved by the institutional review board at the Renmin University of China, but the ethics approval number has not been publicly released. All procedures performed in the study involving human participants were in accordance with the ethical standards of the institutional or national research committee and with the 1964 Helsinki declaration and its later amendments or comparable ethical standards. All participants provided written informed consent. Details of informed consent were stored by the Institute of Gerontology and National Survey Research Center at Renmin University of China.

## Results

### Descriptive Analysis

Table 1 shows the results of descriptive statistics of the sample. The overall level of mental health among older adults was relatively low, with an average score of 2.3122 (SD 0.3439), and only 22.38% (1488/6648) of the older adults who were aged ≥60 years had access to the internet.

Regarding other characteristics, the average age of the sample was 70.8719 (SD 7.0914) years; 49.79% (3310/6648) were male individuals, and 69.66% (4631/6648) were married. The majority of the respondents (5158/6648, 77.59%) had social insurance, 51.04% (3393/6648) lived in a rural area, 36.55% (2430/6648) had only a primary school education, and only 24.94% (1658/6648) were employed. Moreover, the respondents’ level of self-rated health was relatively high, with an average score of 3.3206. In addition, 87.58% (5822/6648) owned 1 home, 37.68% (2505/6648) resided in the eastern region, and 36.61% (2434/6648) lived in the central region.

### Results of Simple Regression

Before the empirical analysis, we conducted a multicollinearity test. The variance inflation factor was far below the critical value of 10, with a mean of 1.30 and a maximum of 1.64. Therefore, multicollinearity did not exist in our data.

Table 2 shows the results of ordinary least squares (OLS) regression. The subgroup analyses showed that older adults who were younger, married, lived in a rural area, had a high level of education, owned more homes, and reported better self-rated health had a higher level of mental health than their counterparts (column 4 of Table 2). However, we found that sex and social insurance were entirely unrelated to the mental health of older adults. In particular, the level of mental health of older adults residing in the western region was significantly lower than that of older adults residing in the central and eastern regions, probably because the central and eastern regions are economically developed, which plays a positive role in improving mental health among older adults.

**Table 2:** Linear regression of the impact of internet use on mental health (n=6648)^a^.

Variable	Model 1, β^b^ (SE)	Model 2, β (SE)	Model 3, β (SE)
Internet use	0.1952^c^ (0.009)	0.118^c^ (0.011)	0.075^c^ (0.011)
Sex	N/A^d^	−0.011 (0.008)	−0.006 (0.008)
Age	N/A	−0.002^c^ (0.006)	−0.003^c^ (0.001)
Marital status	N/A	0.062^c^ (0.009)	0.061^c^ (0.009)
Hukou (household registration)	N/A	−0.022^e^ (0.009)	−0.048^c^ (0.010)
Education	N/A	0.029^c^ (0.004)	0.025^c^ (0.005)
Social insurance	N/A	−0.003 (0.010)	−0.009 (0.010)
Work status	N/A	−0.026^e^ (0.010)	−0.003 (0.010)
Home ownership	N/A	0.058^c^ (0.012)	0.045^c^ (0.012)
Self-rated health	N/A	0.080^c^ (0.005)	0.077^c^ (0.005)
Residing in eastern region	N/A	N/A	0.165^c^ (0.011)
Residing in central region	N/A	N/A	0.037^c^ (0.010)

^a^*R*^2^ for model 1: 0.0558; model 2: 0.0886; model 3: 0.1553.

^b^β is a regression coefficient.

^c^*P*<.001.

^d^N/A: not applicable.

^e^*P*<.01.

### Endogenous Treatment: Instrumental Variables Approach

There may be an endogeneity problem between internet use and the mental health of older adults. Therefore, the 2-stage least squares (2SLS) method was used to reduce the bias caused by the endogeneity problem in the regression results. Theoretically, effective instrumental variables must be uncorrelated with random disturbances. Meanwhile, they must be highly correlated with endogenous variables. On the one hand, internet use is related to the provincial internet penetration rates; on the other hand, the internet penetration rates are not related to the health of older adults at the microindividual level.

The estimation results of the instrumental variable model are reported in Table 3. We used the 2SLS method, which is usually used to analyze the validity of the instrumental variables [41]. First, we estimated the effect of provincial internet penetration rates on internet use. Second, we estimated the effect of internet use on mental health by regression. For a single instrumental variable, *F* statistics values of <10 are thought to suggest a problem of weak instruments [42]. In this study, the *F* statistics value implied by this first-stage regression was 260.52, which allayed any concerns about weak instruments.

**Table 3:** The treatment of endogeneity: instrumental variable model (n=6648)^a^.

Variable	Results of the first-stage regression	Results of the second-stage regression
Instrumental variable (provincial internet penetration rate), β^b^ (SE)	0.0103^c^ (1.39)	N/A^d^
Internet use, β (SE)	N/A	0.2131^e^ (2.90)
Control variables	Yes	Yes

^a^Adjusted *R*^2^: results of the first-stage regression—0.3203 and results of the second-stage regression—0.0721; *F* statistics value: results of the first-stage regression—260.52.

^b^β is a regression coefficient.

^c^*P*<.001.

^d^N/A: not applicable.

^e^*P*<.01.

The regression results of the full sample showed that the regression coefficient of the impact of provincial internet penetration rates on the mental health of older adults was 0.0103, which was significant at the 1% level. Next, the second-stage regression coefficient of the impact of internet use on the mental health of older adults was 0.2131. The results show that internet use had a signiﬁcant positive impact on mental health. Thus, the relationship between internet use and the health of older adults was further veriﬁed.

### Results of Robustness Analysis

To obtain the net effect of internet use on the mental health of older adults, propensity score matching (PSM) was selected to test the robustness of the OLS regression results. We divided the study population into 2 groups: the treatment group (those using the internet) and the control group (those not using the internet). Before using PSM, it was necessary for the samples to pass the balance test, which ensures that no systematic difference exists between the treatment group and the control group after matching except for the independent variables. We used the PSM method to generate a matched comparison group for our analysis. The results of the balance tests are presented in Table 4. Taking the balance test results of radius matching as an example, the SD of all variables was controlled within the desired 6% after matching. All *t* test (2-tailed) values were not signiﬁcant after matching, which showed that after applying the PSM method, the difference between the treatment and control groups was not signiﬁcant, indicating a better matching effect. The results show that the results obtained by using the PSM method were similar to those of random experiments.

We ﬁrst estimated the average treatment effect (ATT) before matching, and the results are shown in Table 5. The ATT before matching was signiﬁcantly higher than that after matching, which meant that if the selection bias was not considered, the inﬂuence of internet use on mental health would be overestimated. We chose the ATTs of different estimation methods for subsequent analysis. In radius matching, the mental health score of the control group was lower than that of the treatment group by 0.01406 and was significant at the level of 1%. The estimation results of k-nearest neighbor matching and Mahalanobis metric matching were the same, indicating that this study is not sensitive to matching methods and has good robustness. Therefore, we can conclude that internet use does have a significant facilitation effect on the mental health of older adults.

**Table 4:** Results of the balance test (obtained by using the radius matching method).

Variable and sample			Treatment group, mean		Control group, mean		Deviation rate (%)		*t* test		*P* value
**Sex**											
	Unmatched	0.511		0.494		3.3		1.130		.26	
	Matched	0.510		0.493		3.5		0.940		.35	
**Age**											
	Unmatched	67.060		71.971		−78.3		−24.580		<.001	
	Matched	67.163		67.388		−3.6		−1.170		.24	
**Marital status**											
	Unmatched	0.829		0.658		39.9		12.790		<.001	
	Matched	0.825		0.822		0.8		0.250		.80	
**Hukou (household registration)**											
	Unmatched	0.798		0.401		88.8		28.660		<.001	
	Matched	0.794		0.793		0.1		0.040		.97	
**Education**											
	Unmatched	1.875		0.953		105.6		35.730		<.001	
	Matched	1.849		1.863		−1.7		−0.440		.66	
**Social insurance**											
	Unmatched	0.878		0.752		32.8		10.410		<.001	
	Matched	0.876		0.868		2.1		0.640		.52	
**Work status**											
	Unmatched	0.168		0.273		−25.5		−8.280		<.001	
	Matched	0.169		0.171		−0.5		−0.160		.88	
**Home ownership**											
	Unmatched	1.181		0.980		54.5		20.030		<.001	
	Matched	1.162		1.181		−5.1		−1.260		.21	
**Self-rated health**											
	Unmatched	3.557		3.252		36.4		12.130		<.001	
	Matched	3.545		3.530		1.9		0.530		.59	

**Table 5:** The average treatment effect (ATT) of internet use on mental health.

Matching method			Treatment group		Control group		ATT value^a^		SD		*t* test
ATT before matching			2.4638		2.2685		0.1953		0.0098		19.86^b^
**ATT after matching**											
	Radius matching	2.4601		2.3195		0.1406		0.0147		9.53^b^	
	K-nearest neighbor matching (k=4)	2.4615		2.3222		0.1393		0.0161		8.63^b^	
	Mahalanobis metric matching	2.4607		2.3245		0.1362		0.0141		9.64^b^	

^a^Treatment group−control group.

^b^*P*<.001.

### Sensitivity Analysis

We also conducted a sensitivity analysis to further test the robustness of the results presented in Table 2. We used the command *rbounds* in Stata (StataCorp LLC) to conduct the *Rosenbaum bounds* analysis, which has been commonly used in existing studies for sensitivity analysis. In the Rosenbaum approach, the sensitivity analysis aims to ensure that no significant hidden biases exist, and Γ (gamma) is an important index that can indicate the level of sensitivity of the study; Γ=2 is a generally used threshold value to claim that the study is free of hidden bias [43].

The results of the sensitivity analysis are displayed in Table 6. Г ranges from 1 to 2.4. When Г increases to 2.4 (>2, which is the threshold value), we can observe a significant sensitivity. Therefore, the sensitivity analysis shows that the results presented in Table 2 are robust, which can partly support that the PSM analysis results are reliable.

**Table 6:** Sensitivity analysis (the results were obtained by using the radius matching method).

Gamma (Γ)	Significance levels			Hodges-Lehmann point estimates			95% CI
	Minimum	Maximum	Minimum		Maximum		
1	<.001	<.001	0.152		0.152	0.133 to 0.170	
1.2	<.001	<.001	0.123		0.180	0.104 to 0.199	
1.4	<.001	<.001	0.099		0.204	0.079 to 0.223	
1.6	<.001	<.001	0.077		0.225	0.058 to 0.244	
1.8	<.001	<.001	0.059		0.243	0.039 to 0.262	
2	<.001	<.001	0.042		0.259	0.022 to 0.278	
2.2	.004	<.001	0.027		0.273	0.007 to 0.292	
2.4	.09	<.001	0.013		0.285	−0.006 to 0.305	

### Results of Heterogeneity Analysis

The impact of internet use on the mental health of older adults may be affected by demographic factors. To further examine the effect of internet use on mental health under various factors, we selected variables such as age, hukou, and education to test heterogeneity.

Internet use had a significant facilitation effect on the mental health of older adults aged 60 to 70 years (OLS, β=.1000; *P<*.001) but it had no significant effect on older adults aged 70 to 80 years and 81-100 years (OLS, β=−.0036; *P*=.87; β=−.0291; *P*=.60). In hukou conditions, the association between internet use and the self-rated health of older adults for both rural and urban samples was also signiﬁcantly positive (OLS, β=.0661; *P<*.001; β=.0882; *P<*.001). However, for those in urban areas, the effect was much smaller. This may be because under the urban-rural dual structure, there are relatively few public and entertainment products and services provided in rural areas. In terms of education level, internet use had a significant facilitation effect on those who listed *primary school and* secondary school, as well as individuals with high school education and above (OLS, β=.0826; *P<*.001; β=.1415; *P<*.001). However, it had no significant effect on uneducated older adults (OLS, β=−.0519; *P=*.11).

### Mechanism Analysis

Previous studies have often used stepwise regression, the Sobel-Goodman test, and the bootstrap method to estimate the mediating effect. Although questions have been raised about the stepwise regression method, some scholars insist that if the total effect is significant, the indirect effect is more explanatory [44].

To explore whether participation in voluntary activities is an intermediary factor driving the relationship between internet use and the mental health of older adults, we constructed the OLS model. Model 4 controlled all other variables, with participation in voluntary activities as an independent variable; model 5 added participation in voluntary activities to model 4; and model 6 added internet use to model 5. Table 7 shows the estimation results of mediating effects. Model 4 shows that internet use had a significant impact on participation in voluntary activities (coefficient=0.0794; *P*<.01). Model 5 shows that participation in voluntary activities was significantly and positively correlated with mental health (coefficient=0.059; *P*<.01). Model 6 shows all positive coefficients and was significant at a 1% level, indicating that internet use significantly affected the mental health of older adults (coefficient=0.0727; *P*<.01). Therefore, the mediating effect of participation in voluntary activities on internet use and the mental health of older adults was significant.

**Table 7:** Estimation results of mediating effects^a^.

Variable	Model 4 (participation in voluntary activities)	Model 5 (mental health)	Model 6 (mental health)
Internet use, β^b^ (SE)	0.0794^c^ (0.014)	N/A^d^	0.0707^c^ (0.011)
Participation in voluntary activities, β (SE)	N/A	0.0529^c^ (0.009)	0.0487^c^ (0.009)
Control variables	Yes	Yes	Yes

^a^*R*^2^ for model 4: 0.1214; model 5: 0.1534; model 6: 0.1584.

^b^β is a regression coefficient.

^c^*P*<.001.

^d^N/A: not applicable.

### Moderating Effect

The test of the moderating effect is based on the method proposed by Wen et al [45]. The results presented in Table 8 suggest that compared with internet nonuse, internet use can significantly improve the mental health of older adults. Subjective age was also found to be significantly and positively associated with mental health. The results demonstrate that subjective age moderates the relationship between internet use and mental health. The positive coefficients of the 2 interaction terms indicated that the positive effect of internet use on mental health was substantially accentuated by the subjective age of older adults.

**Table 8:** Results of the moderation effect analysis (n=6648)^a^.

Variable	Model 7	Model 8
Internet use, β^b^ (SE)	1.049^c^ (0.136)	0.749^c^ (0.141)
Subjective age, β (SE)	N/A^d^	0.043^c^ (0.004)
Internet use×subjective age, β (SE)	N/A	0.050^c^ (0.010)
Control variables	Yes	Yes

^a^*R*^2^ for model 5: 0.120; model 6: 0.136.

^b^β is a regression coefficient.

^c^*P*<.001.

^d^N/A: not applicable.

To further explore the association between internet use and mental health, simple slopes were tested at moderate (M), low (M−1 SD), and high (M+1 SD) levels of subjective age. The relationship was strongest when the levels of subjective age were high (b=0.11, SE 0.01; *P*<.001) compared with both moderate (b=0.06, SE 0.01; *P*<.001) and low (b=0.01, SE 0.01; *P*=.55) levels of subjective age. Specifically, for participants with low subjective age scores, internet use was not related to mental health, whereas for participants with high subjective age scores, internet use was signiﬁcantly associated with mental health. In particular, the simple slopes tests revealed significant positive effects of internet use on mental health that increased as the levels of subjective age increased.

## Discussion

### Principal Findings

The aim of this study was to better explore the relationship between internet use and mental health among Chinese older adults through several complementary methods, such as OLS regression, the 2SLS method, and PSM based on CLASS 2018 data. After endogenous and robustness tests, the results conﬁrmed the robustness of the conclusion that internet use has a signiﬁcant positive impact on mental health. Moreover, this study also examined the different associations between internet use and the health of older adults by age, hukou, and education. In particular, participation in voluntary activities acted as an intermediary between internet use and mental health. The results are discussed in detail in the following paragraphs.

First, internet use and smartphone use can significantly improve the mental health of older adults, which is in line with prior studies by Keane et al [46] and Chang and Im [47]. This may be partly because information and resources on the internet are more abundant, which helps older adults enrich their lives and maintain regular contact with family, friends, and other social network members. Thus, older adults are less prone to depression and show a high level of mental health.

Second, higher mental health levels were found for older adults aged 60 to 70 years who used the internet, whereas no signiﬁcant differences were found for older adults aged >70 years. In addition, the increasing health effect of internet use is more apparent among older adults in rural areas than among those in urban areas. This may be because the internet is commonplace for urban residents, whereas it is still a novelty in rural areas because the development and popularization of the internet started late in rural areas. Therefore, the abundance of information and resources on the internet had a strong attraction for older adults in rural areas. Regarding education level, internet use had a significant facilitation effect on those who listed *primary school and above*. However, it had no significant effect on uneducated older adults, probably because older adults with a higher level of education have a high level of internet skills, and thus they benefit more from the use of the internet.

Third, our ﬁndings demonstrated that participation in voluntary activities plays the role of a mediator between internet use and mental health. On the one hand, internet use will promote participation in voluntary activities. More speciﬁcally, older adults may use the internet as a medium to obtain information regarding voluntary activities, which could further encourage greater participation in voluntary activities [48]. On the other hand, our ﬁndings revealed that participation in voluntary activities could promote the level of mental health among older adults. The aforementioned finding has been evidenced in previous studies [49]. In addition, we further uncovered that subjective age moderated the relationship between internet use and mental health, and younger subjective age helped to enhance the positive effect of internet use on the mental health of older adults.

On the basis of the aforementioned empirical results, we offer the following suggestions on how to improve the mental health of older adults through internet use:

1. Steps should be taken to improve older adults’ attitudes toward internet use. Given that internet technology is thought to be more challenging for older adults to learn [50], they may fail to accept the convenience brought by the internet. In this regard, a targeted training service to enhance the ability of older adults to use the internet will help them become familiar with the various applications (eg, health management, leisure, and social contact), which in turn will bridge the digital divide and help them achieve active aging. Meanwhile, relevant authorities should offer courses to older adults on how to distinguish quality health information from inaccurate, misleading, or fraudulent material.
2. The government should help to create an older adult–friendly environment by building activity centers and increasing public service ﬁscal expenditures. Furthermore, social resources should be mobilized for investment in the older adult care industry.
3. Local communities should organize various forms of voluntary activities for older adults and publicize them widely through the internet to stimulate the interest of older adults in participation in voluntary activities. The low cost and convenience of web-based volunteering information dissemination facilitate older adults to deepen their understanding of volunteering and strengthen their sense of responsibility, inner effectiveness, and public interest. At the same time, the rich information regarding voluntary activities also creates more opportunities for older adults to contact the organizations concerned and participate in these activities.

This study enriches and expands on previous research and contributes to the literature in 3 ways. Previous studies mostly studied the correlation between internet use and mental health from a single perspective. In comparison with these studies, we used an innovative research perspective: we divided internet use into 4 dimensions (whether to use the internet, the frequency of internet use, the number of web-based activities, and smartphone use), providing a multidimensional consideration of internet use connotation. Meanwhile, the endogeneity problem of the regression model was eliminated by selecting an instrumental variable, which made the estimation results more scientific. More importantly, our ﬁndings provide evidence for the ﬁrst time that participation in voluntary activities acts as an important mediating variable between internet use and the mental health of older adults. This not only provides empirical support for the theory that internet use improves the health status of older adults but also supports the promotion of policies on active aging.

However, this study also has some limitations. Because of the presence of variables in secondary data, only participation in voluntary activities was treated as a mediating variable. Given the absence of the broader variables of economic participation, family participation, and political participation, it is difficult to accurately reflect the mediating effect of active aging on internet use and mental health. However, these limitations will provide research directions for future in-depth research.

### Conclusions

On the basis of a national sample of Chinese older adults, this study established a new conceptual framework to explain the mechanism of how internet use affects the mental health of older adults under the mediating role of participation in voluntary activities. This study unveiled that internet use directly affected mental health among older adults, and participation in voluntary activities played an intermediary role between internet use and mental health. In addition, a substantial positive effect of the use of the internet on mental health among older adults was moderated by subjective age.

## Abbreviations

2SLS: 2-stage least squares
ATT: average treatment effect
CLASS: China Longitudinal Aging Social Survey
OLS: ordinary least squares
PSM: propensity score matching

## Data Availability

Restrictions apply to the availability of these data. Data were obtained from the National Survey Research Center at the Renmin University of China with the permission of the National Survey Research Center at the Renmin University of China.

## References

[1] Chen K, Chan AH (2014). Predictors of gerontechnology acceptance by older Hong Kong Chinese. Technovation.

[2] Zhu Y, Zhou Y, Long C, Yi C (2021). The relationship between internet use and health among older adults in China: the mediating role of social capital. Healthcare (Basel).

[3] Coget JF, Yamauchi Y, Suman M (2002). The Internet, social networks and loneliness. IT Soc.

[4] Nie NH, Hillygus DS (2002). The impact of internet use on sociability: time-diary findings. IT Soc.

[5] Gardner PJ, Kamber T, Netherland J (2012). “Getting turned on”: using ICT training to promote active ageing in New York City. J commun Inform.

[6] Yang H, Wu Y, Lin X, Xie L, Zhang S, Zhang S, Ti S, Zheng X (2021). Internet use, life satisfaction, and subjective well-being among the elderly: evidence from 2017 China General Social survey. Front Public Health.

[7] Grossman M (1972). On the concept of health capital and the demand for health. J Polit Econ.

[8] Grossman M, Culyer AJ, Newhouse JP (2000). The human capital model. Handbook of Health Economics.

[9] Cangelosi PR, Sorrell JM (2014). Use of technology to enhance mental health for older adults. J Psychosoc Nurs Ment Health Serv.

[10] Hunsaker A, Hargittai E, Piper AM (2020). Online social connectedness and anxiety among older adults. Int J Commun.

[11] Yuan H (2021). Internet use and mental health problems among older people in Shanghai, China: the moderating roles of chronic diseases and household income. Aging Ment Health.

[12] Lee HY, Kim J, Sharratt M (2018). Technology use and its association with health and depressive symptoms in older cancer survivors. Qual Life Res.

[13] Wilson J (2000). Volunteering. Annu Rev Sociol.

[14] Wilson J (2012). Volunteerism research: a review essay. Nonprofit Volunt Sect Q.

[15] Hank K, Stuck S (2008). Volunteer work, informal help, and care among the 50+ in Europe: further evidence for 'linked' productive activities at older ages. Soc Sci Res.

[16] Nie NH, Erbring L (2002). Internet and society: a preliminary report. Internet Soc.

[17] Mukherjee D (2010). Participation of older adults in virtual volunteering: a qualitative analysis. Ageing Int.

[18] Jin YA, Zhao MH (2019). Internet use and the active aging of Chinese elderly: a study based on 2016 China longitudinal aging social survey. Popul J.

[19] Filsinger M, Freitag M (2018). Internet use and volunteering: relationships and differences across age and applications. Voluntas.

[20] Fernández-Ballesteros R, Dolores Zamarrón M, Angel Ruíz M (2001). The contribution of socio-demographic and psychosocial factors to life satisfaction. Ageing Soc.

[21] Musick MA, Wilson J (2003). Volunteering and depression: the role of psychological and social resources in different age groups. Soc Sci Med.

[22] Li Y, Ferraro KF (2005). Volunteering and depression in later life: social benefit or selection processes?. J Health Soc Behav.

[23] Guo Q, Bai X, Feng N (2018). Social participation and depressive symptoms among Chinese older adults: a study on rural-urban differences. J Affect Disord.

[24] Li Y, Liu M, Miyawaki CE, Sun X, Hou T, Tang S, Szanton SL (2021). Bidirectional relationship between subjective age and frailty: a prospective cohort study. BMC Geriatr.

[25] Uotinen V, Rantanen T, Suutama T (2005). Perceived age as a predictor of old age mortality: a 13-year prospective study. Age Ageing.

[26] Barrett AE, Montepare JM (2015). "It's About Time": applying life span and life course perspectives to the study of subjective age. Annu Rev Gerontol Geriatr.

[27] Seifert A, Wahl H (2018). Young at heart and online? Subjective age and internet use in two Swiss survey studies. Educ Gerontol.

[28] Eastman JK, Iyer R (2005). The impact of cognitive age on internet use of the elderly: an introduction to the public policy implications. Int J Consum Stud.

[29] Wu L, Zhang LQ (2022). The levels and function of digital technology in relieving the loneliness of the elderly. J Central China Normal Univ.

[30] Teuscher U (2009). Subjective age bias: a motivational and information processing approach. Int J Behav Dev.

[31] Keyes CL, Westerhof GJ (2012). Chronological and subjective age differences in flourishing mental health and major depressive episode. Aging Ment Health.

[32] Westerhof GJ, Barrett AE (2005). Age identity and subjective well-being: a comparison of the United States and Germany. J Gerontol B Psychol Sci Soc Sci.

[33] Gao X (2020). Internet use, social cohesion and urban residents' voluntary service participation. Mod Econ Res.

[34] Aalbers T, Baars M, Rikkert MO (2011). Characteristics of effective Internet-mediated interventions to change lifestyle in people aged 50 and older: a systematic review. Ageing Res Rev.

[35] Tang D, Lin Z, Chen F (2020). Moving beyond living arrangements: the role of family and friendship ties in promoting mental health for urban and rural older adults in China. Aging Ment Health.

[36] Li C, Jiang S, Li N, Zhang Q (2017). Influence of social participation on life satisfaction and depression among Chinese elderly: Social support as a mediator. J Community Psychol.

[37] Wang Y (2020). Study on the effect of smartphone use on the subjective health of the elderly: base on the China Longitudinal Aging Social Survey of 2016. Popul Dev.

[38] Chai YF, Guo S (2020). How old is "old": a new idea to promote voluntary work involvement among older adults in China. J Northwest Univ.

[39] Hubley AM, Hultsch DF (2016). The relationship of personality trait variables to subjective age identity in older adults. Res Aging.

[40] China Longitudinal Aging Social Survey.

[41] Chyi H, Mao S (2011). The determinants of happiness of China’s elderly population. J Happiness Stud.

[42] Sovey AJ, Green DP (2011). Instrumental variables estimation in political science: a readers' guide. Am J Pol Sci.

[43] Lin DY, Psaty BM, Kronmal RA (1998). Assessing the sensitivity of regression results to unmeasured confounders in observational studies. Biometrics.

[44] WEN Z, YE B (2014). Analyses of mediating effects: the development of methods and models. Adv Psychol Sci.

[45] Wen ZL, Hou JT, Zhang L (2005). Comparison and application of regulatory effect and mediating effect. J Psychol.

[46] Keane MC, Roeger LS, Allison S, Reed RL (2013). e-Mental health in South Australia: impact of age, gender and region of residence. Aust J Prim Health.

[47] Chang SJ, Im E (2014). A path analysis of Internet health information seeking behaviors among older adults. Geriatr Nurs.

[48] Räsänen P, Kouvo A (2007). Linked or divided by the web?: internet use and sociability in four European countries. Inf Commun Soc.

[49] Van Willigen M (2000). Differential benefits of volunteering across the life course. J Gerontol B Psychol Sci Soc Sci.

[50] Hussain D, Ross P, Bednar P, Rossignoli C, Virili F, Za S (2018). The perception of the benefits and drawbacks of internet usage by the elderly people. Digital Technology and Organizational Change: Reshaping Technology, People, and Organizations Towards a Global Society.

